# Delirium in Hospitalized Older Adults: A Narrative Review

**DOI:** 10.7759/cureus.109991

**Published:** 2026-05-31

**Authors:** Janani Jeyakanthan, German Corso, Benyame Woldetsadik, Simonia Kotilo, Olubusola Esan

**Affiliations:** 1 Internal Medicine, South Texas Health System, McAllen, USA; 2 Child and Adolescent Psychiatry and Pediatric Psychiatry, Tropical Texas Behavioral Health, Harlingen, USA; 3 Emergency Medicine, Raleigh General Hospital, Beckley, USA; 4 Internal Medicine, Raleigh General Hospital, Beckley, USA

**Keywords:** confusion assessment method, delirium, geriatric delirium, hospitalized older adults, prevention, risk factors

## Abstract

Delirium is a common and serious neuropsychiatric syndrome in hospitalized elderly patients, characterized by acute disturbances in attention, awareness, and cognition. It affects a significant proportion of older adults admitted for acute medical illness and an even greater proportion of those undergoing major surgical procedures, such as hip fracture repair or cardiac surgery. Delirium is independently associated with prolonged hospitalization, institutionalization, functional decline, long-term cognitive impairment, and increased mortality. Despite its prevalence and clinical impact, delirium remains frequently underrecognized, particularly in its hypoactive form, which often presents with subtle symptoms such as lethargy, withdrawal, and reduced responsiveness. Delayed recognition can contribute to preventable complications and poorer outcomes in vulnerable elderly patients.

This narrative review examines literature published between 2005 and 2026 on delirium in hospitalized older adults. It explores the pathophysiology, risk factors, clinical presentation, and diagnostic frameworks used to identify delirium. It also summarizes current guideline-based strategies for the prevention and management of delirium, including both pharmacologic and non-pharmacologic approaches. Improving recognition and management of delirium in hospitalized elderly patients remains an important clinical priority. Greater integration of guideline-based screening and prevention strategies into routine hospital practice may help reduce the incidence of delirium and mitigate its long-term consequences.

## Introduction and background

Delirium is an acute neuropsychiatric syndrome characterized by disturbances in attention, awareness, and cognition that develop over a short period of time and fluctuate throughout the course of the day. Among hospitalized older adults, delirium represents a syndrome of major clinical importance due to its high prevalence, preventable nature, and association with significant adverse outcomes. Estimates suggest delirium affects approximately 23% of elderly medical inpatients, with rates rising to approximately 31% in intensive care unit settings and as high as 75% in mechanically ventilated patients [1,2]. The implications of delirium extend well beyond the hospitalization itself. Patients who experience delirium are two to three times more likely to die within 6-12 months, 60% more likely to experience functional decline, and nearly twice as likely to require institutionalization compared to those without delirium [3]. Even after apparent resolution of the acute episode, up to 65% of older adults demonstrate persistent cognitive deficits at 12 months, highlighting the long-term neurologic consequences associated with this condition [4]. Despite this substantial burden, delirium remains under-recognized in clinical practice, particularly in its hypoactive form. Hypoactive delirium, which predominates in elderly patients, often presents with subtle symptoms such as lethargy, decreased responsiveness, or withdrawal, and may easily be mistaken for depression or fatigue [5]. As a result, many cases are diagnosed late or missed entirely. This is partly due to time constraints, limited staff training, and a lack of consistent screening protocols across hospital units. Clinical guidelines from the American Geriatrics Society (AGS) and the National Institute for Health and Care Excellence (NICE) emphasize the importance of early recognition, routine screening, and preventive strategies to reduce the incidence and complications of delirium in hospitalized patients [6,7].

Older adults are particularly vulnerable to delirium due to age-related declines in physiologic reserve, a higher prevalence of chronic disease, and reduced cognitive resilience. Hospitalization introduces additional precipitating factors, including acute illness, infections, medication exposure, metabolic disturbances, sleep disruption, and environmental stressors. In many cases, delirium develops through the interaction between these acute triggers and underlying vulnerabilities such as dementia, frailty, or sensory impairment. Because several of these risk factors are potentially modifiable, improving recognition and understanding of delirium represents an important opportunity to reduce preventable morbidity and improve outcomes among hospitalized elderly patients. However, despite extensive research on delirium in this population, important gaps remain in the consistent implementation of evidence-based screening protocols in routine clinical practice, as well as in the development of targeted pharmacologic therapies for prevention and treatment.

This narrative review aims to examine current literature on delirium in hospitalized older adults. This paper explores the epidemiology, pathophysiology, risk factors, clinical presentation, and evidence-based strategies for the prevention and management of delirium in this population. Its purpose is to better understand the factors that contribute to the development of delirium during hospitalization and to identify clinical approaches that may reduce its incidence and associated complications. A narrative review approach was chosen rather than a systematic review because the available literature on delirium in hospitalized elderly patients spans a wide range of study designs, including observational studies, clinical guidelines, and expert consensus statements. This format allows for the integration of diverse sources of evidence and provides a broader overview of current understanding and clinical practice.

## Review

Research methods

This narrative review examines recent literature and expert perspectives on the prevention and management of delirium in hospitalized older adults, drawing upon findings from multiple study designs and healthcare contexts.

Types of Studies and Participants

Observational cohort studies, systematic reviews and meta-analyses, randomized controlled trials, and clinical guidelines. Older patients (≥ 65 years) who were diagnosed with delirium in a variety of clinical settings, including medical wards, surgical units, and ICUs.

Search Strategy

Databases searched included PubMed, Embase, and the Cochrane Library. Search terms were: delirium, hospitalized older adults OR elderly OR geriatric patients, risk factors AND delirium, delirium prevention, delirium diagnosis OR confusion assessment method, delirium management OR treatment, postoperative delirium, and hospital-acquired delirium. Relevant MeSH terms and Boolean operators were used to optimize the search strategy and identify relevant studies.

Intervention Type and Outcome Measures

Prevention and management of delirium using pharmacologic and non-pharmacologic approaches. Incidence of delirium, hospital length of stay, cognitive decline, institutionalization, and mortality in elderly hospitalized patients.

Language and Timeframe

Only studies published in English were included. Studies published between September 2005 and January 2026 were reviewed.

Study Selection

Articles were selected using a two-stage review process. In the first stage, titles and abstracts were screened to assess relevance to the care and management of delirium in hospitalized older adults. In the second stage, full-text articles were reviewed to confirm eligibility according to predefined inclusion and exclusion criteria. Reference lists of included studies and recent reviews were also examined to identify additional relevant articles. Current clinical guidelines were obtained from the AGS and the NICE. Studies focusing exclusively on pediatric populations, adults younger than 65 years, outpatient delirium, or non-hospital settings were excluded.

Quality Assessment

The quality of included studies was critically appraised using established appraisal frameworks appropriate to study design, including CASP checklists, the Cochrane Risk of Bias tool for randomized trials, and the Newcastle-Ottawa Scale for observational studies. As this is a narrative review, no quantitative synthesis, pooled effect sizes, or meta-analysis was performed or intended. This review is not designed to meet PRISMA criteria for systematic reviews or meta-analyses.

Pathophysiology

Delirium arises from a multifactorial interplay between baseline vulnerabilities and acute precipitating insults. In the elderly, age-related neurodegeneration, microvascular injury, and reduced synaptic plasticity diminish cognitive reserve, rendering the brain more susceptible to stress [8].

Neurotransmitter imbalance is a central mechanism. Acetylcholine deficiency is strongly implicated, and medications with anticholinergic properties are consistently associated with delirium. Conversely, excess dopamine contributes to hyperactive features such as hallucinations and agitation, while serotonin, GABA, and glutamate dysregulation further disrupt cortical networks [9].

Inflammation plays a central role in delirium, particularly in older adults whose blood-brain barriers are already compromised by age and chronic disease. Systemic insults such as infection or surgery trigger tissue macrophages and cerebrovascular endothelial cells to release inflammatory mediators, including IL-1β, TNF-α, and prostaglandins, which reach the brain through both direct penetration and indirect activation of resident microglial cells. In a vulnerable aging brain, these microglia are already primed; when activated, they amplify the inflammatory signal rather than contain it, releasing additional cytokines and reactive oxygen species that disrupt neurotransmission and synaptic function [10].

This is precisely where age becomes such a critical factor. An older brain is not simply a younger brain that has been around longer. Rather, it has accumulated decades of microvascular wear and tear, neuronal loss, and shrinking synaptic reserve. When inflammation hits, there is far less buffer. A physiologic insult that a younger patient might clear without any lasting neurologic consequence can, in an elderly patient, tip an already strained system into dysfunction that does not fully resolve. This vulnerability is not incidental. It is structural, and it goes a long way toward explaining why cognitive decline after delirium is so much more common, and so much harder to reverse, in older adults than in any other age group [11].

**Figure 1:**
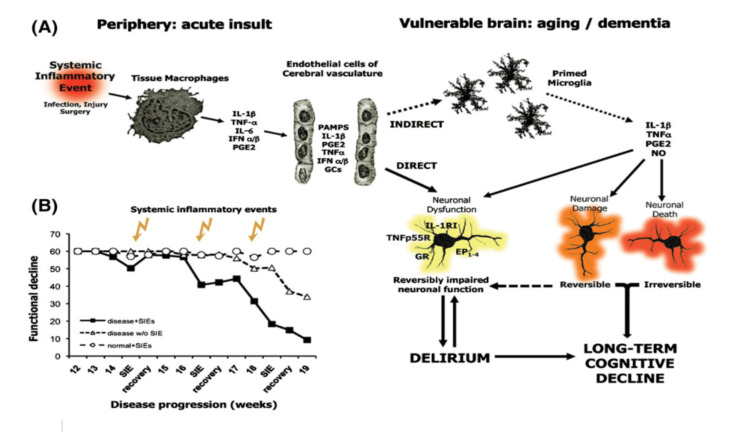
(A-B) Neuroinflammatory pathway linking systemic illness to delirium and long-term cognitive decline. Source: Reproduced from Maldonado [11]. Permission obtained from John Wiley & Sons, Inc. via Copyright Clearance Center.

What makes this process particularly concerning is its cumulative nature. Maldonado proposed that repeated systemic inflammatory events produce neuronal dysfunction that becomes progressively less reversible with each episode, offering a biological explanation for why patients who survive delirium so often fail to return to their cognitive baseline (Figure 1) [11].

Circadian rhythm disruption is another contributing factor, and one that the hospital environment actively worsens. Repeated nocturnal interruptions for vitals and medications, persistent artificial lighting, and ambient noise disrupt sleep patterns and destabilize the arousal systems that regulate attention and cognition. In older patients with limited physiologic reserve, even a few nights of disrupted sleep can significantly lower the threshold for delirium [8].

Neuroimaging has helped clarify what this looks like at the level of the brain. Delirium is associated with cortical hypoperfusion and reduced connectivity within attentional and salience networks [10], suggesting that the cognitive dysfunction seen in delirium reflects not just neurotransmitter imbalance and neuroinflammation, but a broader failure of network-level integration. Notably, this impaired connectivity may itself sustain the delirium episode. A brain with compromised perfusion and disrupted circuitry is less capable of recovering from the same stressors that triggered the episode to begin with.

Risk factors for delirium in hospitalized older adults

Delirium in hospitalized older adults is a multifactorial syndrome that typically develops when baseline vulnerability is combined with an acute precipitating stressor. Older age, pre-existing dementia, mild cognitive impairment, frailty, malnutrition, sensory impairment, and polypharmacy all increase susceptibility by reducing cognitive and physiologic reserve. In the inpatient setting, these underlying vulnerabilities make patients less able to compensate for acute insults such as infection, dehydration, electrolyte abnormalities, hypoxia, pain, urinary retention, immobility, and sleep disruption. In particular, dementia is one of the strongest risk factors for delirium. Cognitively impaired patients have little reserve to draw on, and even seemingly minor shifts in their medical status or surroundings can be enough to push them over the threshold [12].

Polypharmacy, defined as the use of more medications than are clinically necessary, is common in adults aged ≥65 years and is particularly concerning when those medications have central nervous system effects. Benzodiazepines warrant special caution, as they can worsen confusion, impair attention, and precipitate or prolong delirium, especially in older adults with limited reserve. Opioids and anticholinergic medications are also commonly implicated, as both can alter mental status and increase the risk of delirium. In this context, delirium should be viewed not as an isolated event but as the result of an interaction between patient vulnerability and hospital-related triggers. For this reason, early identification of high-risk patients is important, as preventive strategies such as medication review, adequate hydration, and mobilization support can reduce the likelihood of delirium developing during hospitalization [13].

The concept of delirium risk is well captured by the vulnerability-stress paradigm, in which delirium is seen as the result of the interplay between inherent vulnerabilities of a patient and a stressful stimulus or insult. Under the vulnerability-stress paradigm, a very vulnerable patient - for instance, a person with advanced dementia, frailty, or sensory impairment - can suffer from delirium owing to a small physiologic stressor, while a low-vulnerability patient would need to be exposed to several insults at the same time before succumbing to delirium [12]. From a clinical perspective, the paradigm implies that attention should not be directed towards finding one particular reason why delirium occurs, but rather should be focused on mitigating the risk by addressing all possible causes. For example, validated instruments such as HARP (Hospital Admission Risk Profile) and CAM-ICU (Confusion Assessment Method for the ICU) can assist with this task [6].

Among hospitalized older adults, delirium is often precipitated by acute infection, metabolic abnormalities, uncontrolled pain, dehydration, recent surgery, and sleep disruption. Acute infections such as pneumonia, sepsis, and urinary tract infections are common triggers of delirium in older adults [14]. However, clinicians should be cautious when diagnosing asymptomatic bacteriuria, as treating patients without a true infection may result in unnecessary antibiotic use. Nevertheless, early recognition of at-risk patients is essential for prevention.

Clinical presentation and diagnosis

Delirium is clinically classified into hyperactive, hypoactive, and mixed subtypes. Hyperactive delirium presents with agitation, restlessness, and hallucinations, whereas hypoactive delirium, which accounts for up to 70% of elderly cases, presents with withdrawal, decreased responsiveness, and lethargy, often leading to misdiagnosis as depression or fatigue. Mixed delirium, the most common subtype, is characterized by fluctuating features of both hyperactive and hypoactive presentations within the same episode [15].

Diagnosis requires recognition of characteristic features and structured application of validated criteria. The DSM-5 criteria remain the diagnostic gold standard and require the presence of a disturbance in attention and awareness with an acute onset and a fluctuating course. An additional disturbance in cognition must also be present, which may involve memory, orientation, language, visuospatial ability, or perception. These disturbances must not be better explained by a preexisting or evolving neurocognitive disorder and must not occur in the context of severely reduced arousal. Finally, there must be evidence that the disturbance is a direct physiological consequence of a medical condition, substance intoxication or withdrawal, or exposure to a toxin [15].

For practical bedside assessment, the CAM is the most validated tool. The CAM translates these criteria into four operationalized features: acute onset with a fluctuating course, inattention, disorganized thinking, and altered level of consciousness. A positive CAM diagnosis requires the presence of both acute onset with a fluctuating course and inattention, plus either disorganized thinking or altered consciousness [16]. Both AGS and NICE endorse CAM for routine screening, with NICE recommending daily monitoring in older adults with acute illness, hip fracture, or ICU admission [6,7].

The most important step in evaluating delirium is identifying an acute change from the patient’s baseline, which requires history from a family member, caregiver, or healthcare staff. This helps differentiate delirium from dementia by clarifying the sudden onset and fluctuating course of symptoms, and it may also point toward the underlying cause (Table 1, as outlined by Inouye et al. [1]). A brief cognitive assessment should be performed using tools such as the Mini-Cog or Montreal Cognitive Assessment. If time is limited, simple bedside attention tasks, such as reciting the months of the year backward or performing serial 7s, can serve as a quick and effective screen [1]. Given the high risk of complications and mortality, any suspected or unclear case, including patients who are lethargic or unable to fully participate, should be managed as delirium until proven otherwise.

**Table 1:** Initial workup for evaluation of delirium. Source: Adapted from Inouye et al. [1]. Copyright permission obtained via the Copyright Clearance Center. Note: This workup should be individualized based on clinical context. Not all listed investigations are required in every patient.

Evaluation of Delirium	
History	Baseline cognitive function and recent changes in mental status (e.g., family, staff). Recent changes in condition, new diagnoses, review of systems. Review all current medications, including over-the-counter medications and herbal remedies. Review any new medications and potential drug interactions. Review alcohol and benzodiazepine use. Assess for pain and discomfort (e.g., urinary retention, constipation, thirst).
Vital signs	Include temperature, oxygen saturation, fingerstick glucose. Postural vital signs as needed.
Physical and neurological examination	Search for signs of occult infection, dehydration, acute abdomen, deep vein thrombosis, and other acute illnesses. Assess for sensory impairments. Search for focal neurological changes and meningeal signs.
Targeted laboratory evaluation (selected tests based on clues from history and physical)	Based on history and physical examination, consider: Laboratory tests: CBC, electrolytes, calcium, glucose, renal function, liver function, thyroid function, urinalysis, urine, blood and sputum cultures, drug levels, toxicology screen, ammonia level, vitamin B12 level, and cortisol level. Arterial blood gas. Electrocardiography. Chest X-ray. Lumbar puncture is reserved for evaluation of fever with headache, meningeal signs, or suspicion of encephalitis.
Targeted neuroimaging (selected patients)	Assess focal neurological changes, since stroke can present as delirium. Suspicion of encephalitis for temporal lobe changes. History or signs of head trauma.
Electroencephalography (selected patients)	Evaluate for occult seizures. Differentiate psychiatric condition from delirium.

Complications and prognosis

Delirium carries a significant complication burden, both during hospitalization and well beyond it. In the acute setting, delirious patients are two to four times more likely to experience falls, aspiration pneumonia, and unintended removal of catheters or IV lines. The clinical response to agitation, physical restraints, and sedative medications often makes things worse, prolonging immobility and deepening the very confusion it was meant to address [17].

Long-term complications are equally concerning. Delirium is associated with a twofold increased risk of institutionalization, a threefold risk of developing dementia, and a 15%-30% rise in one-year mortality [3]. The financial burden reflects this trajectory. Leslie and colleagues found that adjusted healthcare costs per day survived were more than two and a half times higher in patients who developed delirium, with costs attributable to delirium ranging from $16,000 to over $64,000 per patient in the year following hospitalization. This translates to an estimated national burden of $38 to $152 billion annually [18]. The majority of these costs were driven by inpatient and nursing home care, reinforcing that the consequences of a delirium episode do not end at discharge.

The BRAIN-ICU study, conducted by Pandharipande et al. at Vanderbilt University and published in the New England Journal of Medicine, illustrated this further [17]. One in four critically ill patients had cognitive impairment at 12 months comparable to mild Alzheimer's disease, and one in three showed deficits resembling moderate traumatic brain injury, in a cohort where only 6% had any baseline cognitive impairment before ICU admission [19]. Longer delirium duration was independently associated with worse global cognition and executive function at follow-up, regardless of age, sedative exposure, or comorbidity burden. The proposed mechanisms include delirium-associated neuroinflammation and neuronal apoptosis driving brain atrophy and white matter disruption, both of which are independently linked to cognitive decline. Notably, patients with pre-existing dementia or reduced cognitive reserve are at greatest risk for these long-term consequences, as they have less capacity to compensate for delirium-associated neuronal injury.

For many older adults, delirium can permanently alter their cognitive trajectory even when the acute episode appears to have fully resolved. Even previously healthy patients may not return to baseline. A single hospitalization complicated by delirium can mark the beginning of a cognitive decline that they can never fully recover from [3,19].

Prevention and management

Because no single treatment reliably prevents delirium, management depends on a multimodal, largely nonpharmacologic approach. The most well-studied model is the Hospital Elder Life Program (HELP), which targets six modifiable risk factors: cognitive impairment, immobility, dehydration, sensory loss, and sleep disruption, through daily reorientation, early mobilization, fluid encouragement, hearing and vision aids, and structured sleep protocols. HELP has been shown to reduce delirium incidence by 53% (OR 0.47, 95% CI 0.37-0.59) and falls by 42% (OR 0.58, 95% CI 0.35-0.95), while shortening hospital stays by over a day [20]. Additionally, implementation of HELP has also been associated with significant cost savings, with estimates ranging from $1,600 to $3,800 per patient in hospital costs and more than $16,000 per person-year in long-term care costs, making it a clinically and economically efficient intervention [20]. Simple environmental measures help too: natural light during the day, less noise at night, and keeping clocks and calendars visible go a long way toward maintaining a patient’s sense of time and place. Family presence is underutilized but valuable, providing familiar reassurance that no clinical protocol can fully replicate [21].

Pharmacologic options are limited and should be used cautiously. Antipsychotics such as haloperidol may be indicated for severe agitation when there is an immediate risk to the patient or others. However, due to their known adverse effects, they should generally be reserved as a last resort rather than used routinely. The evidence is clear: they do not prevent delirium, shorten its duration, or improve cognitive outcomes [6,9,16]. In older adults, particularly those with dementia, they carry real risks, including QT prolongation, extrapyramidal side effects, oversedation, and an increased risk of cerebrovascular events, which is why the FDA issued a black box warning [22]. Both AGS and NICE recommend against routine use. Anticholinergic agents, including diphenhydramine, bladder antimuscarinics, and tricyclic antidepressants, carry well-documented deliriogenic risk and should be avoided or substituted in older hospitalized patients wherever clinically feasible, consistent with AGS Beers Criteria recommendations [23]. Benzodiazepines are similarly problematic unless the delirium is driven by alcohol or benzodiazepine withdrawal specifically. Among pharmacologic agents being studied for delirium prevention, ramelteon has shown the strongest evidence, demonstrating efficacy in a network meta-analysis (OR 0.07, 95% CI 0.01-0.66). Dexmedetomidine has shown benefit specifically in non-cardiac surgical populations. Melatonin, however, has not demonstrated significant benefit over placebo in multiple studies [24]. Given these differences, the choice of pharmacologic agent should be guided by the clinical context and the available evidence for each individual drug.

The ABCDEF bundle in an ICU setting includes several items such as assessing and managing pain, both spontaneous awakening and breathing trials, choice of analgesia and sedation, delirium screening and prevention, early mobility, and family engagement. The ABCDEF bundle was specifically designed and validated for ICU settings and should not be directly extrapolated to general medical or surgical wards without appropriate adaptation. In non-ICU settings, multicomponent programs such as HELP remain the preferred framework for delirium prevention. The implementation of the ABCDEF bundle has resulted in significant decreases in the incidence of delirium, number of days on a ventilator, and length of stay in the ICU. In addition, the ABCDEF bundle provides a good framework for preventing delirium using a team approach. Family involvement, as one part of the bundle, should be highlighted as a measure for preventing delirium in any hospital setting. Familiar faces, conversations, and constant human interaction can provide much-needed cognitive stimulation. In addition, nurse training should also be highlighted. Nurses and other health professionals who can detect the early signs of delirium, especially hypoactive delirium, can report their observations more effectively [25].

Ultimately, the most effective approach to delirium is a preventive one: recognizing at-risk patients before delirium develops, systematically targeting modifiable risk factors, and using medications only when truly necessary.

## Conclusions

While delirium is now widely recognized as a medical emergency in older adults, important gaps remain in its recognition and management. Hypoactive delirium is particularly difficult to detect because it does not present dramatically. Instead, patients may appear quiet, withdrawn, or less responsive, which can easily be mistaken for depression, fatigue, or expected post-operative recovery. It is important to acknowledge that the evidence for pharmacological treatments in delirium is still limited and that there is considerable variability across studies. Thus, the recommendations in this review should be interpreted carefully and not taken as definitive guidelines. When it comes to implementing HELP, it is not as simple as just introducing a new protocol. It requires strong clinical leadership, institutional support, and a shift in organizational culture, all of which can be difficult to achieve in busy hospital settings. Improving delirium detection will likely depend on integrating validated screening tools such as the CAM and CAM-ICU into routine nursing assessments, not only in intensive care units but also across general medical and surgical wards.

Future research should extend beyond diagnosis to focus on identifying reliable biomarkers, further clarifying the biological link between delirium and long-term dementia risk, and developing targeted interventions for high-risk populations. These include patients with pre-existing dementia, post-surgical patients, and those receiving palliative care - groups that carry a disproportionate burden of delirium but remain underrepresented in clinical studies. One practical way to improve screening rates is to incorporate delirium assessments such as the CAM into nurses’ existing shift routines rather than treating it as a separate task, which can be time-consuming in already busy clinical settings. Ultimately, incorporating these practices into routine clinical care is essential for improving outcomes and supporting older adults in preserving their independence and quality of life.
